# Validation of the MTXPK Tool in Predicting Glucarpidase Utilization Among Patients With Delayed Methotrexate Clearance: A Retrospective Study

**DOI:** 10.7759/cureus.101794

**Published:** 2026-01-18

**Authors:** Kathie Wu, Chandrakala Dadeboyina, Gwen Hua

**Affiliations:** 1 Hematology/Oncology, Geisinger Medical Center, Danville, USA

**Keywords:** glucarpidase, high dose methotrexate, kidney toxicity, leukemia, lymphoma, mtxpk

## Abstract

Introduction

Glucarpidase, a recombinant bacterial enzyme, can treat patients with methotrexate (MTX) toxicity. Identifying appropriate administration of glucarpidase can be challenging. MTXPK.org, a web-based clinical decision tool, maps projected MTX clearance for individual patients based on demographics and serum MTX levels to identify patients at the highest risk for toxicity who might benefit from glucarpidase administration. Here, we report our single institution experience with the predictive value of the MTXPK web tool in guiding glucarpidase administration.

Methods

Retrospective data collection conducted from 2000 through 2023 of patients admitted for high-dose methotrexate (HDMTX) who received glucarpidase therapy. Data required for utilization of the MTXPK tool were collected (age, sex, race, height, weight, MTX dose, creatinine, serum MTX, albumin, and the presence of pleural effusion). Data was input into the MTXPK tool to plot a curve of MTX clearance rate for each patient compared to the population predicted curve. Patient MTX clearance rates that fell below the predicted population rates were candidates for glucarpidase.

Results

Thirteen patients were included for review, with ages ranging from four months to 72 years, with an average of 47.7 years. Nine patients (69%) were male, and four (31%) were female. Twelve were Caucasian patients (92.3%), and one was a Black patient (7.7%). Average height and weight were 66.6 inches and 83.6 kg, respectively, with an average body surface area (BSA) of 1.99 kg/m^2^. Six patients (46.2%) had lymphoma, two (15.4%) had sarcoma, and five (38.5%) had leukemia. One hundred percent of MTXPK predictions matched published glucarpidase administration guidelines. MTXPK predicted that only eight patients (61.5%) should have received glucarpidase, indicating five (38.5%) of patients were administered the drug unnecessarily.

Conclusion

Clinicians should utilize MTXPK as an objective tool to assist in clinical decision-making in patients with delayed MTX clearance with the goal of limiting unnecessary use of glucarpidase and avoiding unintended outcomes of MTX toxicity.

## Introduction

High-dose methotrexate (HDMTX), doses ≥500 mg/m^2^, treats a broad range of malignancies [1]. Methotrexate (MTX) interferes with folate metabolism and reduces cell proliferation, but crystallizes in the renal tubules and causes nephrotoxicity. Impaired renal function causes decreased drug clearance and numerous systemic toxicities.

Measures to prevent HDMTX toxicity include IV hydration, urine alkalinization, leucovorin rescue, and monitoring serum creatinine and plasma MTX concentrations. Maintaining urine pH ≥7 increases MTX solubility and prevents drug precipitation. In patients who fail the above rescue techniques, alternative measures to enhance MTX clearance are recommended.

Glucarpidase, a recombinant bacterial enzyme, rapidly hydrolyzes MTX into noncytotoxic metabolites and reduces serum levels by 98% within 30 minutes [2]. The FDA approved glucarpidase for use within 60 hours of MTX infusion in patients who develop HDMTX-induced nephrotoxicity or delayed MTX excretion [3].

MTXPK.org, a web-based tool, utilizes age, sex, height, weight, creatinine, MTX dose, and plasma concentration to generate a patient's projected drug excretion curve compared to a population predicted curve [4]. This provides an objective tool to help clinicians decide which patients will benefit from glucarpidase administration. We evaluated the use of MTXPK as an objective tool in our institution to determine appropriate administration of glucarpidase in patients with predicted delayed MTX clearance and to limit use in patients who are not at high risk to limit medication cost and toxicity.

## Materials and methods

**Figure 1:**
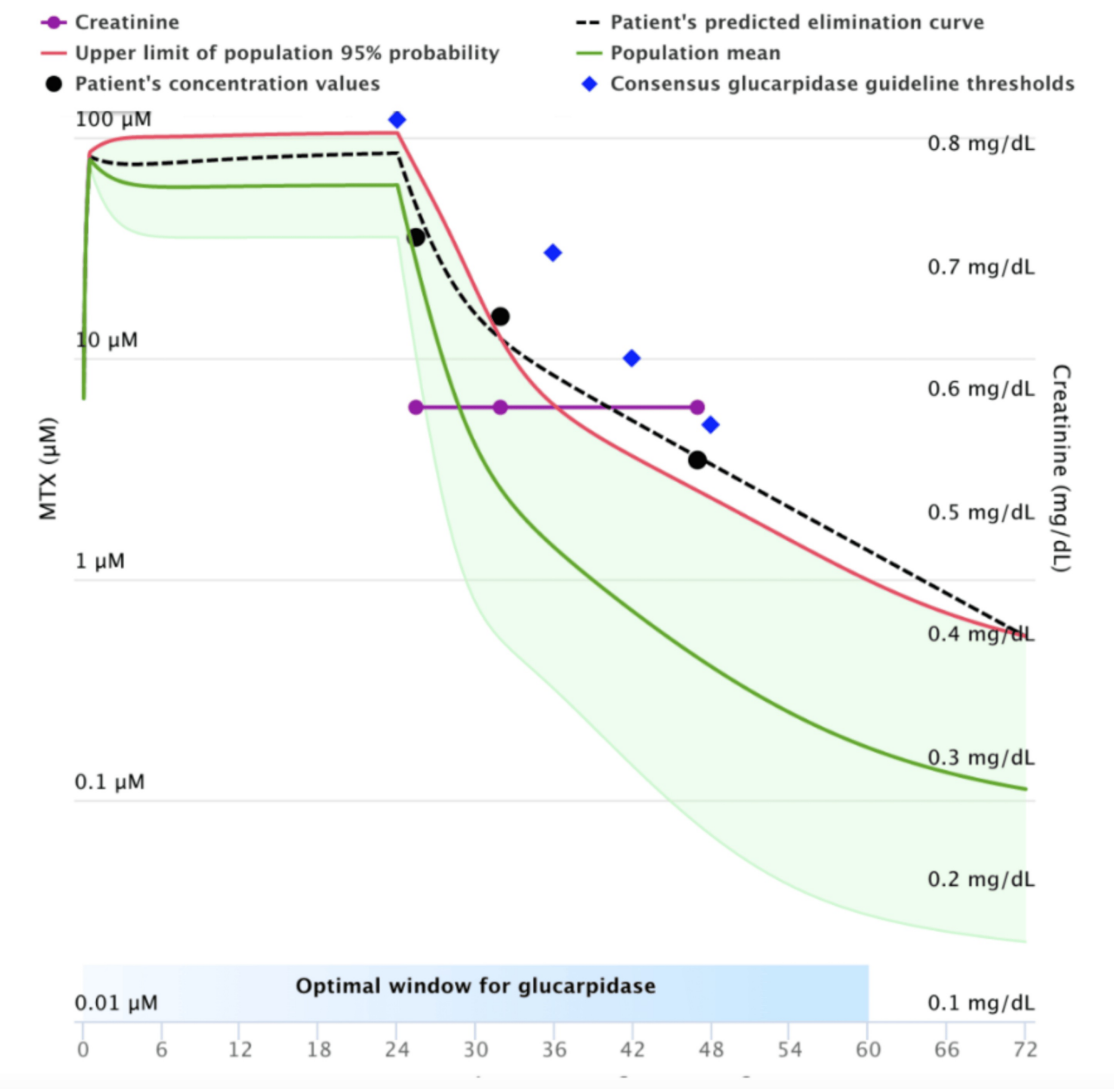
MTXPK Depicting No Indication for Glucarpidase The graph displays the patient's individual methotrexate (MTX) concentration over time (black dashed line) as compared to the population-predicted curve (blue diamonds), which is the expected median concentration profile for a typical patient given the same dose and characteristics (age, sex, height, weight, creatinine). In this graph, the patient’s individual concentrations fall close to and below the population curve, indicating the patient is eliminating the drug as expected/slightly better than average.

**Figure 2:**
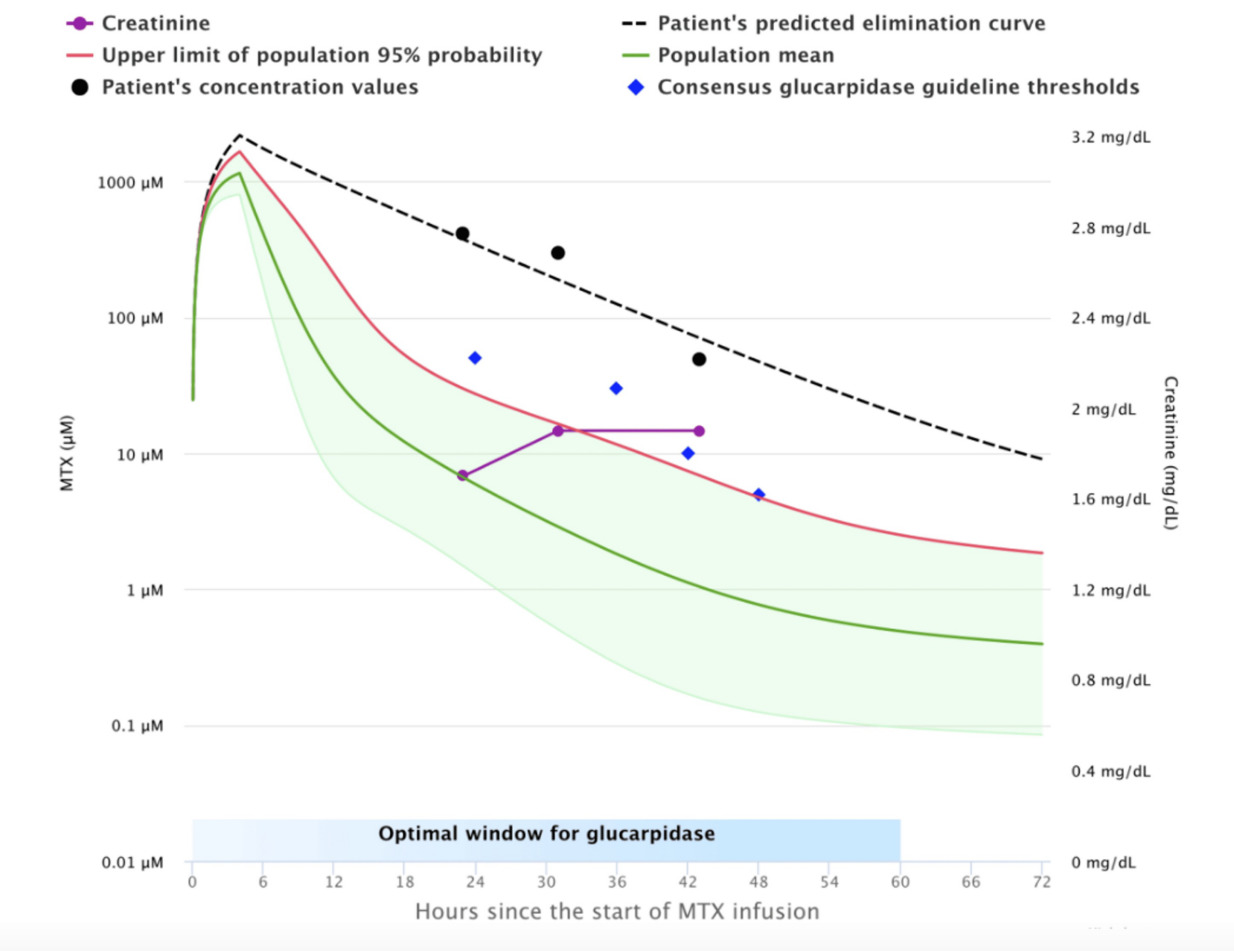
MTXPK Depicting Need for Glucarpidase Administration The graph displays the patient's individual methotrexate (MTX) concentration over time (black dashed line) as compared to the population-predicted curve (blue diamonds), which is the expected median concentration profile for a typical patient given the same dose and characteristics (age, sex, height, weight, creatinine). In this graph, the patient’s individual concentrations fall above the population curve, indicating the patient is eliminating the drug more slowly than average. This can lead to prolonged high concentrations, increasing the risk of MTX toxicity.

Retrospective review conducted from January 1, 2000, to January 1, 2023, of patients who had inpatient admission for HDMTX (infusion or bolus) and received glucarpidase rescue. Data required for utilization of the MTXPK tool were collected, including patient age, sex, race, height, weight, MTX dose, creatinine, serum MTX levels, albumin, and presence of pleural effusion. The collected patient data was input into the MTXPK tool to plot a curve of MTX clearance rate for each patient compared to the population predicted curve. Patients whose MTX clearance rates fell below the predicted population rates were deemed as candidates for glucarpidase administration (Figure 1), whereas patients who had MTX clearance rates that were within the upper limits of excretion did not meet criteria for glucarpidase administration (Figure 2).

## Results

Thirteen patients were included for review, all having received glucarpidase rescue. Ages ranged from four months to 72 years, with an average of 47.7 years overall. Nine (69%) of patients were males, and four (31%) were females. Twelve of the 13 were Caucasian patients (92.3%), and one was a Black patient (7.7%). Average height and weight were 66.6 inches and 83.6 kg, respectively, with an average body surface area (BSA) of 1.99 kg/m^2^. Six patients (46.2%) had lymphoma, two (15.4%) had sarcoma, and five (38.5%) had leukemia (Table 1).

**Table 1:** Patient Demographics BSA: body surface area

Variable	Value
Total patients, n	13
Female	4
Male	9
Age (mean ± SD)	47.7 ± 21.7
Height, cm (mean ± SD)	169.1 ± 31.7
Weight, kg (mean ± SD)	86.3 ± 31.5
BSA, m2 (mean ± SD)	1.99 ± 0.57
Caucasian individuals	12
Black individuals	1
Lymphoma	6
Sarcoma	2
Leukemia	5

One hundred percent of patients who were identified at high risk for decreased MTX clearance by MTXPK predictions matched the glucarpidase expert guideline thresholds for glucarpidase administration (Table 2).

**Table 2:** Consensus Guideline for the Use of Glucarpidase The table illustrates the current consensus guidelines for the use of glucarpidase therapy based on serum methotrexate (MTX) levels at corresponding time periods after high-dose methotrexate (HDMTX) infusion administration.

Hours After HDMTX Infusion	Serum MTX (µmol)
24	>50
36	>30
42	>10
48	>5

MTXPK predicted that only eight of the 13 patients (61.5%) met guidelines to receive glucarpidase therapy. Five patients (38.5%) who were administered glucarpidase had MTXPK curves that were below the population threshold, indicating that these patients were clearing at or better than average, indicating low risk of MTX toxicity and would not have met consensus guidelines for glucarpidase administration (Table 3).

**Table 3:** Indications for Glucarpidase Administration The table illustrates whether glucarpidase administration was indicated as per MTXPK predictions or consensus guidelines and whether glucarpidase was ultimately given.

Patient	Glucarpidase per MTXPK (Y/N)	Glucapidase per Guidelines (Y/N)	Drug Given (Y/N)
1	Y	Y	Y
2	N	N	Y
3	Y	Y	Y
4	Y	Y	Y
5	Y	Y	Y
6	N	N	Y
7	Y	Y	Y
8	N	N	Y
9	Y	Y	Y
10	N	N	Y
11	Y	Y	Y
12	N	N	Y
13	Y	Y	Y

None of the 13 patients who received glucarpidase therapy was noted to develop grade 3 or 4 nephrotoxicity. Seven patients had 48- to 72-hour post-MTX creatinine levels either at or better than baseline. The remaining six patients had no more than grade 1 acute kidney injury (AKI), defined as an increase in creatinine of no more than 0.3 mg/dL or a rise of 1.5- to 2-fold above baseline creatinine.

## Discussion

MTX administered at high doses can penetrate the blood-brain barrier and treat a variety of malignancies, including CNS lymphoma, sarcoma, and leukemia in children and adults. Patients who have delayed clearance of MTX are at risk for renal toxicity and life-threatening side effects to the bone marrow, liver, and mucosa [5]. Despite the use of preventative measures such as IV fluids, urine alkalization, and leukocytosis, these toxicities, particularly kidney injury, can occur in up to 12% of patients, and neither hemodialysis nor peritoneal dialysis sufficiently removes the drug from circulation [2,3].

Since the approval of glucarpidase by the FDA in 2012, we had an effective antidote for MTX toxicity. Once administered, this recombinant bacterial enzyme can cleave 99% of circulating MTX into inactive metabolites within 15-30 minutes of administration [5]. However, due to ambiguity in the drug label indications, the proper utilization of glucarpidase was challenging. A consensus guideline for glucarpidase use was published in 2017 [6], attempting to illustrate set time points with corresponding MTX levels at which glucarpidase administration should be considered (Table 2). In clinical practice, when plasma MTX levels were obtained at time points other than those predetermined in the study (24, 36, and 48 hours), application of the algorithm became challenging.

With the development of MTXPK [4], we now have a web-based clinical decision tool that helps identify the appropriate rate of MTX clearance based on a population model, and the patients who fall below the population curve are at high risk for MTX toxicity. The tool allows the user to input a series of demographic information, including patient age, calculated BSA, dose of MTX administered, infusion time, serum albumin, and the presence of pleural effusions, which causes drug accumulation. Compared to the glucarpidase consensus guidelines, which required plasma MTX levels to be drawn at the above mentioned predetermined time points in order to be interpretable, the MTXPK tool allows flexibility to input any time, measured as hours post MTX administration, along with serum creatinine and corresponding serum MTX level. The tool then graphs the individual patient data against a preexisting pharmacokinetic model to generate a predicted patient drug clearance curve as compared to a population curve for the same demographic. This then allows users to identify patients who are not clearing MTX at the expected or necessary rate, who require additional intervention with glucarpidase in order to prevent MTX toxicity. This provides an opportunity for earlier identification of potential MTX toxicity and quicker interventions, which translates to better patient outcomes.

Despite this promising drug, studies on the use of glucarpidase are limited. One study looked at 28 cancer centers across the US and found that only 209 patients of 708 with MTX-induced kidney injury were administered glucarpidase, even though the data showed both improved renal and nonrenal outcomes with glucarpidase use in this population [5]. The study does show a small percentage of adverse reactions associated with glucarpidase use, including nausea, diarrhea, and paresthesia. In addition, glucarpidase comes at a high cost, at $27,000 per 1000 unit vial [7]. Compared to standard of care, studies have shown an increased cost of over $20,000 per patients who receive glucarpidase for MTX toxicity, though this was shown to be less expensive per patient than delayed glucarpidase treatment or treatment with hemodialysis [8]. This illustrates the need for timely and appropriate selection of patients for glucarpidase use, which can be accomplished with the use of the MTXPK tool.

Limitations of this study included a small sample size and a retrospective study design, which relies on documentation present within the chart and has a limited ability to assess adverse reactions to glucarpidase, underestimating safety outcomes. Racial diversity was also limited, with only one Black individual and otherwise predominantly Caucasian, which may impact generalizability given the impact of social background on patient outcomes. Given the lack of a true control group, as all patients already received glucarpidase, this limits the true predictive value and comparative outcomes to patients who did receive intervention. There are potential confounding factors, including hydration status, leucovorin rescue timing and dosing, baseline renal function, comorbidities, drug interactions, and institutional treatment protocols that are unable to be fully captured or adjusted. Given that MTXPK.org models rely on population-based pharmacokinetic models, this does represent patient populations at extremes such as age, obesity, and critical illness.

## Conclusions

While effective, HDMTX utilization should be closely monitored with care taken to ensure patients are clearing the drug appropriately. In patients who fail standard of care methods to prevent MTX toxicity, consideration should be given to the utilization of glucarpidase therapy. However, due to potential adverse events associated with glucarpidase and the costs of treatment, administration of glucarpidase should still be done judiciously. Only patients who are at high risk for MTX toxicity should receive glucarpidase therapy, and these patients can be effectively identified via the use of the MTXPK web tool.

Our institution's experience showed that MTXPK not only matched with glucarpidase guideline recommendations in 100% of the patients, but also allowed for more flexibility in the hourly monitoring of serum MTX levels than traditional algorithms. We were also able to appropriately identify five patients who were given glucarpidase unnecessarily, highlighting an opportunity for improvement in healthcare utilization.
